# Perceived Harm of Snus and Smokeless Tobacco Among US Adults

**DOI:** 10.1001/jamanetworkopen.2025.43267

**Published:** 2025-11-19

**Authors:** Alexander Persoskie, Carlos Portillo, Claudia C. Ma, Olusola Oniyide

**Affiliations:** 1 Office of Science, Center for Tobacco Products, US Food & Drug Administration, Silver Spring, Maryland

## Abstract

This case series assesses the perceived harm of snus and smokeless tobacco among US adults and whether these products are perceived to be equally or more harmful than cigarettes.

## Introduction

From 2013 to 2019, the proportion of US adults who correctly perceived snus and smokeless tobacco as less harmful than cigarettes declined significantly.^1^ From 2018 to 2019, only 6.4% and 7.0% of US adults perceived snus and smokeless tobacco, respectively, as less harmful than cigarettes.^1^ It is unknown whether these perceptions have changed since October 2019, when the US Food and Drug Administration began issuing modified risk tobacco product orders for specific snus and smokeless tobacco products that authorized those products to be marketed with certain information about lower health risks compared with cigarettes.^2^ We report the perceived harm of snus and smokeless tobacco compared with cigarettes in 2022 and 2023 among adults overall and among adults who use snus and smokeless tobacco.

## Methods

For this analysis, data were obtained from wave 7 of the Population Assessment of Tobacco and Health (PATH) Study,^3,4^ from January 2022 to April 2023. The study was conducted by Westat and approved by the Westat Institutional Review Board. All respondents aged 18 years or older provided informed consent. Consistent with the AAPOR reporting guideline, information on sampling, administration, response rates, instruments, and the Codebook are available in the PATH Study.^3,4^

Participants answered the question, “Is using snus less harmful, about the same, or more harmful than smoking cigarettes?” with the responses less harmful, about the same, more harmful, or don’t know. Participants answered the same question about smokeless tobacco (eg, moist snuff, dip, spit, or chew). The study classified participants as those who used (or did not use) snus, smokeless tobacco, and cigarettes in the past 30 days, based on items about ever having used each product type, having used it in the past 30 days, number of days using it in the past 30 days, last time used, and length of time since last using it.

We conducted statistical analyses in SAS, version 9.4 (SAS Institute Inc). We weighted analyses to produce nationally representative estimates and account for the sampling design. We applied a balanced repeated replication method using the Fay adjustment (0.3).^4^

## Results

The overall weighted sample reflected population demographics in terms of sex, age, and educational attainment (less than high school diploma or General Education Development, high school graduate, some college or associate’s degree, bachelor’s degree or more). Current snus and smokeless tobacco users were mostly male (84.4% [95% CI, 78.0%-89.3%] and 93.7% [95% CI, 91.3%-95.4%], respectively), and current snus users were younger (mean [SE] age, 39.5 [1.1] years) than current smokeless tobacco users (46.0 [0.7]) and adults overall (48.0 [0.05]).

Overall, 7.8% (95% CI, 7.3%-8.3%) and 7.5% (95% CI, 7.1%-8.0%) of US adults correctly perceived snus and smokeless tobacco, respectively, as less harmful than cigarettes (Table 1). These percentages were higher among people who used snus and smokeless tobacco in the past 30 days (Table 1).

**Table 1.** Perceived Harm of Snus and Smokeless Tobacco vs Cigarettes Among US Adults by Tobacco Use Status^a^

Population subgroup	Survey response, No. (% [95% CI])			
	Less harmful	About the same	More harmful	Don’t know
**Perceived harm of snus vs cigarettes**				
Overall (all adults, regardless of tobacco use)	2532 (7.8 [7.3-8.3])	19 071 (67.8 [67.1-68.6])	7935 (23.5 [22.7-24.2])	213 (0.9 [0.7-1.1])
Used snus in past 30 d	68 (30.1 [23.3-37.9])	132 (55.1 [47.5-62.5])	45 (14.8 [10.8-19.9])	0
**Perceived harm of smokeless tobacco vs cigarettes**				
Overall (all adults, regardless of tobacco use)	2347 (7.5 [7.1-8.0])	20 604 (71.7 [71.0-72.4])	6624 (20.0 [19.3-20.6])	175 (0.8 [0.7-1.1])
Used smokeless tobacco in past 30 d	181 (28.9 [23.8-34.6])	436 (63.3 [57.8-68.6])	52 (6.3 [4.3-9.0])	6 (1.5 [0.6-3.6])^b^

^a^ Frequencies are unweighted; percentages are weighted. Survey respondents with missing data on each variable were excluded from any analysis that included that variable. Past 30-day product use was defined using the derived variables R07R_A_P30D_SNUS (for snus) and R07R_A_P30D_SMKLS (for smokeless tobacco).

^b^ This estimate should be interpreted with caution because it has low statistical precision. It is based on a denominator sample size of less than 50, or the coefficient of variation of the estimate or its complement is larger than 30%.

Among people who used smokeless tobacco, those who did not smoke cigarettes were more than twice as likely to perceive smokeless tobacco as less harmful than cigarettes compared with those who smoked cigarettes in the past 30 days (33.9% [95% CI, 27.6%-40.9%] vs 16.4% [95% CI, 10.6%-24.6%]) (Table 2). The same pattern was observed for snus, but it did not reach statistical significance.

**Table 2.** Perceived Harm of Snus and Smokeless Tobacco vs Cigarettes Among US Adults Who Use Each Product Type by Cigarette Smoking Status^a^

Population subgroup	Survey response, No. (% [95% CI])		OR (95% CI)^b^	AOR (95% CI)^c^
	About the same, more harmful, or don’t know	Less harmful		
**Perceived harm of snus vs cigarettes**				
Used snus and smoked cigarettes in past 30 d	96 (76.5 [66.2-84.4])	28 (23.5 [15.6-33.8])	1 [Reference]	1 [Reference]
Used snus but did not smoke cigarettes in past 30 d	81 (64.6 [53.1-74.5])	40 (35.4 [25.5-46.9])	1.78 (0.86-3.68)	1.47 (0.72-3.02)
**Perceived harm of smokeless tobacco vs cigarettes**				
Used smokeless tobacco and smoked cigarettes in past 30 d	183 (83.6 [75.4-89.4])	37 (16.4 [10.6-24.6])	1 [Reference]	1 [Reference]
Used smokeless tobacco but did not smoke cigarettes in past 30 d	311 (66.1 [59.1-72.4])	144 (33.9 [27.6-40.9])	2.61 (1.47-4.65)	2.23 (1.22-4.08)

Abbreviations: AOR, adjusted odds ratio; OR, odds ratio.

^a^ Frequencies are unweighted; percentages are weighted. Survey respondents with missing data on each variable were excluded from any analysis that included that variable. Past 30-day product use was defined using the derived variables R07R_A_P30D_SNUS (for snus), R07R_A_P30D_SMKLS (for smokeless tobacco), and R07R_A_P30D_CIGS (for cigarettes).

^b^ ORs are from a weighted logistic regression of perceived harm (less harmful = 1; about the same, more harmful, or do not know = 0) on cigarette smoking status (did not smoke cigarettes in the past 30 days = 1; smoked cigarettes in the past 30 days = 0), among people who used the product type (snus or smokeless tobacco) in the past 30 days.

^c^ AORs are from the aforementioned weighted logistic regression but were also adjusted for sex, age, and educational attainment (reference category: less than high school diploma or General Education Development).

## Discussion

In 2022 and 2023, over 90% of US adults continued to perceive snus and smokeless tobacco as equally or more harmful than cigarettes. Many people who smoke cigarettes may overlook snus and smokeless tobacco as potential alternative products, while many who use snus or smokeless tobacco may be more likely to initiate smoking because they do not understand the increased health risks. The US Food and Drug Administration aims to provide clear information about the relative risks of various tobacco products^5^ and the continuum of risk^6^ to help US adults better understand these products’ differential risks.

## Notes

Supplement.
Data Sharing Statement
