# Supplementary material for: Perceived Harm of Snus and Smokeless Tobacco Among US Adults
Source: JAMA Netw Open. 2025 Nov 19;8(11):e2543267. doi: 10.1001/jamanetworkopen.2025.43267 (PMC12631493; doi:10.1001/jamanetworkopen.2025.43267)
Supplement: Supplement. — Data Sharing Statement [file jamanetwopen-e2543267-s001.pdf]

Data Sharing Statement

Persoskie. Perceived Harm of Snus and Smokeless Tobacco Among US Adults. *JAMA Netw Open*. Published November 12, 2025. doi:10.1001/jamanetworkopen.2025.43267

Data
Data available: Yes
Data types: Deidentified participant data, Data dictionary
How to access data: The PATH Study data are available via download or application at https://doi.org/10.3886/Series606.
When available: beginning date: 04-08-2024

Supporting Documents
Document types: Informed consent form
How to access documents: Informed consent is available at https://doi.org/10.3886/Series606
When available: beginning date: 04-08-2024

Additional Information
Who can access the data: The PATH Study public-use data files are available via download for access by the general public. The PATH Study restricted use data files are available via application and require completing a Restricted Data Use Agreement. See more information at: https://www.icpsr.umich.edu/web/NAHDAP/studies/36231
Types of analyses: These data are to be used solely for statistical analysis and reporting of aggregated information, and not for the investigation of specific individuals or organizations.
Mechanisms of data availability: Discussed above.
